# Bladder Cancer Exhibiting High Immune Infiltration Shows the Lowest Response Rate to Immune Checkpoint Inhibitors

**DOI:** 10.3389/fonc.2019.01101

**Published:** 2019-10-31

**Authors:** Shen Pan, Yunhong Zhan, Xiaonan Chen, Bin Wu, Bitian Liu

**Affiliations:** ^1^Department of Radiology, Shengjing Hospital of China Medical University, Shenyang, China; ^2^Department of Urology, Shengjing Hospital of China Medical University, Shenyang, China

**Keywords:** bladder urothelial cancer, molecular subtype, single-sample gene set enrichment analysis, weighted gene co-expression network analysis, immune checkpoint inhibitor, immune cell infiltration level, biomarker

## Abstract

**Background:** Bladder urothelial cancer (BLCA) treatment using immune checkpoint inhibitors (IMCIs) can result in long-lasting clinical benefits. However, only a fraction of patients respond to such treatment. In this study, we aimed to identify the relationships between immune cell infiltration levels (ICILs) and IMCIs and identify markers for ICILs.

**Methods:** ICILs were estimated based on single-sample gene set enrichment analysis. The response rates of different ICILs to IMCIs were calculated by combining the ICILs of molecular subtypes in BLCA with the response rates of different molecular subtypes of IMvigor 210 trials to a programmed cell death ligand-1 inhibitor. Weighted gene co-expression network analysis was used to identify modules of interest with ICILs. Functional enrichment analysis was performed to functionally annotate the modules. Screening of key genes and unsupervised clustering were used to identify candidate biomarkers. Tumor IMmune Estimation Resource was used to validate the relationships between the biomarkers and ICILs. Finally, we verified the expression of key genes in molecular subtypes of different response rates for IMCIs.

**Findings:** The basal squamous subtype and luminal infiltrated subtype, which showed low response rates for IMCIs, had the highest levels of immune infiltration. The neuronal subtypes, which showed the highest response rates to IMCIs, had low ICILs. The modules of interest and key genes were determined based on topological overlap measurement, clustering results, and inclusion criteria. Modules highly correlated with ICILs were mainly enriched in immune responses and epithelial–mesenchymal transition. After screening the key genes in the modules, five candidate biomarkers (*CD48, SEPT1, ACAP1, PPP1R16B*, and *IL16*) were selected by unsupervised clustering. The key genes were inversely associated with tumor purity and were mostly expressed in the basal squamous subtype and luminal infiltrated subtypes.

**Interpretation:** Patients with high ICILs may benefit the least from treatment with IMCIs. Five key genes could predict ICILs in BLCA, and their high expression suggested that the response rate to IMCIs may decrease.

## Introduction

Bladder urothelial cancer (BLCA) is one of the 10 most common malignancies worldwide. Systemic platinum-based chemotherapy, introduced nearly 30 years ago, remains the standard of care for untreated patients with inoperable or advanced metastatic BLCA and is associated with a 5-year survival rate of less than or equal to 15% (1). The landscape for BLCA has recently shifted following the approval of therapies targeting the programmed cell death-1 (PD-1)/programmed cell death ligand 1 (PD-L1) axis (2). Anti-immune checkpoint therapy has tremendously expanded our knowledge of the immunobiology of cancer. However, response rates to immune checkpoint inhibitors (IMCIs) are approximately 20% in both the platinum refractory and previously untreated settings (3). Tumor immune cell therapy may be the key to combatting cancer; however, the relationship between immune cell infiltration levels (ICILs) and the therapeutic effects of IMCIs is not clear.

The therapeutic effects of IMCIs are associated with the expression of PD-L1 and tumor-infiltrating lymphocytes (TILs) (4), as well as tumor mutational burden (TMB) (5). Notably, even if TILs are similar, the tumor microenvironment (TME) may still vary, and differences in mixed infiltration vs. septal infiltration may explain these variations (6). Moreover, there are many types of TILs, including CD8^+^ T cells, which kill tumor cells, and regulatory T cells (Tregs), which inhibit CD8^+^ T cells. Subtle changes in the proportions of immune cells can have different effects on tumor progression (7). Overall, tumor escape from immune surveillance and tumor counterattacks against immune cells are extremely complex and multifactorial processes.

Molecular subtypes of muscle invasive bladder cancer (MIBC), classified using The Cancer Genome Atlas (TCGA) data, revealed different ICILs and suggested that the basal squamous subtype and luminal infiltrated subtype can be treated with IMCIs (8). Indeed, these subtypes have been reported to respond to immune checkpoint therapy (9, 10). However, a recent study found that the neuronal subtype, which is associated with a poor prognosis, had the highest response rate after using the PD-L1 inhibitor atezolizumab, and prognosis was significantly better than those of the other four subtypes (11). The complete response (CR) and partial response (PR) of the neuronal subtype reached 100%, whereas those of the other three subtypes, excluding the luminal subtype, did not exceed 20%.

IMCIs may have applications as novel treatments for patients with BLCA. However, the relationships between ICILs and the efficacy of IMCIs have not been elucidated. Accordingly, in this study, we examined that ICILs could be used to predict the therapeutic effects of IMCIs and evaluated candidate biomarkers for ICILs through network analysis and unsupervised clustering.

## Materials and Methods

### Data Sources and Preprocessing

The RNA-sequencing (RNA-seq) results from 433 tissues and 408 cases of human bladder transitional cell carcinoma and papilloma samples, as well as data on the clinical characteristics of the patients, were obtained from TCGA database (portal.gdc.cancer.gov). Five MIBC molecular subtypes based on TCGA data were derived from a study by Robertson et al. (8). The response rates of different molecular subtypes to IMCIs were described by Kim et al. (11). In single-sample gene set enrichment analysis (ssGSEA), we normalized the expression data to Transcripts Per Million after calculating the total lengths of all exons for all transcripts. In weighted gene co-expression network analysis (WGCNA), we normalized the data using R package edgeR and filtered differentially expressed genes (DEGs) with an absolute fold change of >1 and a false discovery rate of <0.05.

### Gene Signatures

A previously described procedure was used to determine the infiltration of immune cells in BLCA (12). We obtained a marker gene set for immune cell types from Bindea et al. (13). To calculate the one-sample gene set enrichment, we used the GSEA program to obtain the absolute enrichment scores from previously validated gene signatures.

### Implementation of ssGSEA

The infiltration levels of immune cell types were quantified by ssGSEA in R package gsva. ssGSEA applies gene signatures expressed by immune cell populations (13) to individual cancer samples. The deconvolution approach used in our study included 24 immune cell types that were involved in innate immunity [natural killer (NK) cells, NK CD56^dim^ cells, NK CD56^bright^ cells, dendritic cells (DCs), plasmacytoid DCs, immature DCs, activated DCs (aDCs), neutrophils, mast cells, eosinophils, and macrophages] and adaptive immunity [B, T, CD8^+^ T, T helper (Th), Th1, Th2, Th17, T gamma delta, T central memory, T effector memory, T follicular helper (Tfh), Tregs, and cytotoxic T cells]. The obtained cytolytic activity (CYT) score obtained from the data set of Rooney et al. (14) consisted of cytolytic genes and was calculated as the geometrical means for perforin 1 and granzyme A. These two key cytolytic effectors are dramatically upregulated upon CD8^+^ T-cell activation and during productive clinical responses to anti-CTLA-4 and anti-PD-L1 immunotherapies; these two molecules are co-expressed in TCGA samples (14). We used the CYT scores to evaluate immune infiltration in different samples.

### WGCNA and Module Preservation

WGCNA was performed using the WGCNA R package (15). Because some genes with no significant changes in expression between samples were highly correlated in WGCNA, genes with the most differential expression were used in subsequent WGCNA. Genes with the highest 25% of DEG variance were selected (16), guaranteeing the heterogeneity and accuracy of bioinformatics statistics for further co-expression network analysis. First, RNA-seq data were filtered to reduce outliers. The co-expression similarity matrix consisted of the absolute values of the correlation between transcript expression levels. A Pearson correlation matrix was constructed for paired genes. We constructed a weighted adjacency matrix using the power function amn = |cmn|β (cmn = Pearson correlation between gene m and gene n; amn = adjacency between gene m and gene n). The parameter β emphasized a strong correlation between genes and penalized a weak correlation. Next, an appropriate β value was selected to increase the similarity matrix and achieve a scale-free co-expression network. The adjacency matrix was then converted into a topological overlap matrix (TOM), which measured the network connectivity of genes defined as the sum of adjacent genes generated by all other networks. Average linkage hierarchical clustering was performed based on TOM-based dissimilarity measurements, and the minimum size (genome) of the gene dendrogram was 30. Through further analysis of modules, we calculated their dissimilarity and constructed module dendrograms.

### Confirmation of Significant Modules

To determine the significance of each module, gene significance (GS) was calculated to measure the correlation between genes and sample traits. Module eigengenes were considered the main components in the principal component analysis of each gene module, and the expression patterns of all genes were summarized as a single feature expression profile within a given module. Next, GS was defined as the log10 conversion of the *p*-value in the linear regression between gene expression and clinical data (GS = lgP). Module significance (MS) was defined as the average GS within the module and calculated to measure the correlation between the module and sample traits. Statistical significance was determined using the relevant *p*-values. In order to increase the capacity of the modules, we selected a cutoff (<0.25) to merge some modules with similar heights. Next, we selected the ICILs previously calculated by ssGSEA and some clinical data for the clinical phenotype. The gene modules associated with the clinical phenotype were then analyzed.

### Key Gene Identification

After selecting modules of interest, we calculated GS and module membership (MM, correlation between the module own genes and gene expression profiles) for each key gene and set their thresholds. In most similar studies, the thresholds for screening key genes in the module were defined as cor. gene MM >0.8 and cor. gene GS >0.2 (17, 18), and we adjusted these values appropriately to suit our study.

### Functional Annotation—FunRich

FunRich (www.funrich.org) is an independent software tool for functional enrichment and interaction network analysis of genes and proteins (19). To explore the biological functions of genes in modules, we used the FunRich enrichment analysis function for analysis.

### Tumor IMmune Estimation Resource (TIMER)

TIMER (cistrome.shinyapps.io/timer) provides a user-friendly web interface for dynamic analysis and visualization of the associations between immune infiltrates and gene expression (20). We used the Gene module to verify the association between genes and immune infiltration. The scatterplots were generated and displayed, showing Spearman's correlation and statistical significance.

### Validation of Key Genes in the Different Molecular Subtypes

Few expression profiling, sequencing, or array data have been published for different prognostic outcomes after treatment with IMCIs. Therefore, according to the different responses of various molecular subtypes to IMCIs, we identified data with molecular subtypes to verify the key genes.

## Results

### Immune Phenotype Landscape in BLCA

**Figure 1:**
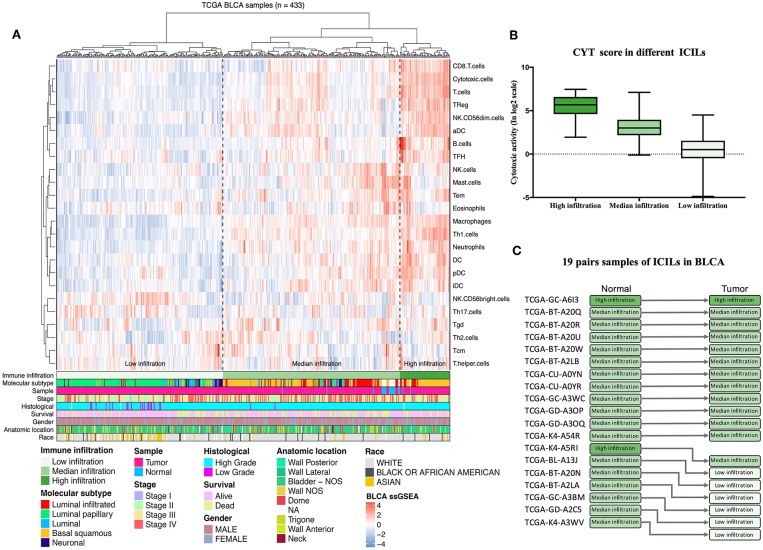
Immune infiltration landscape of BLCA. **(A)** Unsupervised clustering of 408 patients from The Cancer Genome Atlas cohort using single-sample gene set enrichment analysis scores from 24 immune cell types. Molecular subtype, sample type, stage, histological, survival, gender, anatomic location, and race are shown in the lower panel. Hierarchical clustering was performed with Euclidean distance and Ward linkage analyses. Three distinct immune infiltration clusters, termed high infiltration, moderate infiltration, and low infiltration, were defined. **(B)** Relative cytolytic scores (CYT scores) for tumors with high, moderate, and low immune infiltration were clustered according to overall immune cell infiltration. Kruskal–Wallis tests were used for analyses, and results with *P-*values of < 0.0001 were considered significant. Error bars represent the medians, interquartile ranges, and minimum to maximum values. **(C)** The evolution of immune infiltration levels in 19 cases with paracancerous tissues.

Diverse immune cell populations infiltrate the TME and activate or suppress the antitumor response. To assess the spectrum of immune cell infiltration, the ssGSEA (21) approach was utilized to deconvolve the relative abundance of each cell type based on expression profiling data retrieved from the TCGA database. In this analysis, 408 patients with BLCA, for whom transcriptome profiling data and clinical characteristics were available, were included in this study. We found significant heterogeneity in terms of infiltration of numerous immune cell types among the cohort (Figure 1A). To facilitate further characterization, unsupervised clustering was applied to categorize the cohort into three infiltration subgroups, i.e., termed high (*n* = 55), median (*n* = 195), and low (*n* = 183) infiltration.

Considering the concomitant infiltration of activating and suppressive immune cell types, we investigated whether higher ICILs were correlated with elevation of cytotoxic function. To this end, CYT, which can serve as a surrogate index for quantifying the magnitude of the antitumor response, was examined. Patients with high infiltration status showed the highest CYT scores, indicating that cytotoxic function was efficiently elicited in those patients (Figure 1B).

A comparative analysis of 19 cases with normal tissues revealed that most normal tissues showed moderate infiltration. The corresponding cancer tissues showed a partial decrease in the ICILs (Figure 1C). Although the difference in ICILs was minor, we found that this result was related to lack of sufficient post-cluster ICILs grading. After increased grading, the ICILs in all cases were different. Therefore, in the next WGCNA, we analyzed DEGs.

### ICILs of Different Molecular Subtypes

**Figure 2:**
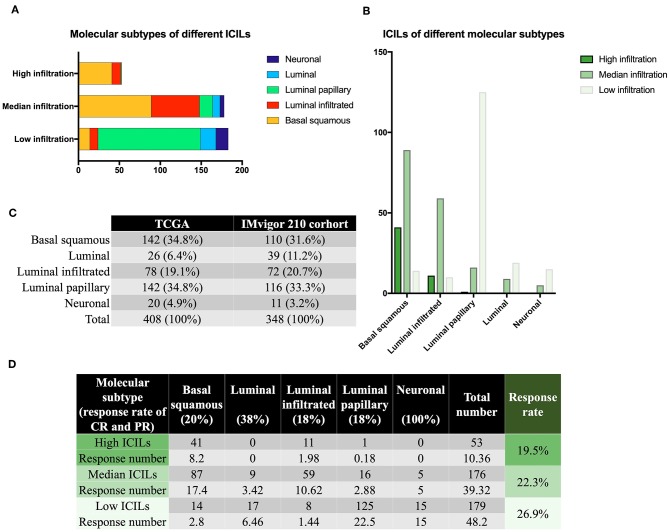
Molecular subtypes and immune infiltration. **(A)** Different ICILs showed variations in the proportions of molecular subtypes. **(B)** ICILs of different molecular subtypes were altered. **(C)** TCGA was similar to the IMvigor 210 trial in terms of total sample size and molecular subtype ratio. **(D)** The low ICIL group had the highest efficacy after receiving IMCIs. High ICILs had the lowest efficiency. ICILs, immune cell infiltration levels; IMCIs, immune checkpoint inhibitors.

Although there were few cases of the neuronal subtype, the high response rate to IMCIs was interesting. There were only 20 samples of neuronal subtypes; 15 had low ICILs, and 5 had moderate ICILs. Thus, although 75% of cases of the neuronal subtype had low ICILs, we could not assume that low ICILs were responsive to IMCIs because most low ICILs were observed for the luminal-papillary subtype, which had a low response rate to IMCIs (Figure 2A). In addition to the neuronal subtype, the luminal subtype showed a better response rate than the other three subtypes, and 68 and 32% of cases of this subtype had low and moderate ICILs, respectively (Figure 2B). There were only three molecular subtypes showing high ICILs, and their response rates to IMCIs were all low; the basal squamous subtype and the luminal infiltrated subtype accounted for 98% of cases. Thus, these findings showed that high ICILs were associated with low response rate to IMCIs.

### CR and PR of Different ICILs

Based on the CR and PR ratios of different molecular subtypes, we calculated the response rates of different ICILs to IMCIs. The ratio of CR to PR was derived from the IMvigor 210 cohort study (11), which included 348 cases, and the proportion of each molecular subtype was similar to that of the 408 cases from the TCGA database (Figure 2C). Finally, we calculated the ratio of CR to PR for different ICILs in TCGA in combination with the ratio of each molecular subtype (Figure 2D). Although the response rates of different ICILs did not exceed 30%, the response rate to IMCIs increased with the decrease in ICILs. The percentage of low ICILs was 7% higher than that of high ICILs.

### WGCNA: Identification of the Most Significant Modules and Genes

**Figure 3:**
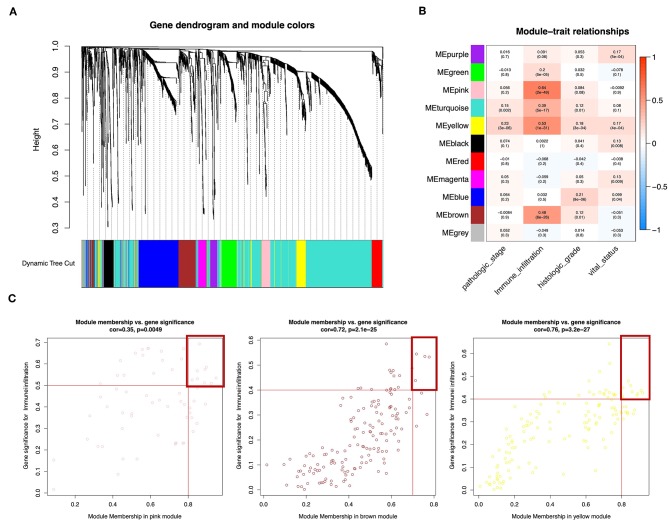
Weighted gene co-expression network of bladder urothelial cancer. **(A)** Identification of a co-expression module in bladder urothelial cancer. The branches of the cluster dendrogram correspond to the 11 different gene modules. Each piece of the leaves on the cluster dendrogram corresponds to a gene. **(B)** Correlations between the gene module and clinical traits. The correlation coefficient in each cell represents the correlation between the gene module and the clinical traits, which decreased in size from red to blue. The corresponding *P*-values are also shown. **(C)** Scatter plot of module eigengenes in pink, brown, and yellow modules. The red line indicates the screening threshold. The burgundy box identifies the key genes for each module.

Wgcna was performed to construct a gene co-expression network for identification of biologically significant gene modules and elucidation of genes associated with ICILs. Eliminating outlier samples (Supplementary Figure 1A), the 8510 DEGs with the highest 25% of variance by cluster analysis were placed in a module. In this study, we selected β = 3 (scale-free *R*^2^ = 0.957) as a soft threshold to ensure a scale-free network (Supplementary Figure 1B) and obtained 11 modules without merging for subsequent analysis (Figure 3A).

To analyze the relationships between the modules and ICILs of the samples, we used MS as the overall gene expression level of the corresponding module to calculate the correlations with clinical phenotypes. The pink module was most significantly associated with the ICILs, with a correlation close to 0.65. In addition, the brown and yellow modules exhibited relatively high positive correlations with immune infiltration (Figure 3B). Thus, we chose these modules as the modules of interest and used them for subsequent analyses.

To determine the importance of genes in the module and the correlations between gene expression and ICILs, we appropriately adjusted the screening thresholds for MM and GS to select key genes. We identified 9 key genes (*ACAP1, CD48, CXCR4, GYPC, IL16, NCKAP1L, PPP1R16B, SEPT1*, and *WIPF1*, cor.MM >0.8 and cor.GS > 0.5) in the pink module, 4 key genes (*PARP12, TAP1, TAP2*, and *TYMP*, cor.MM >0.7 and cor.GS >0.4) in the brown module, and 10 key genes (*COL6A2, CTHRC1, EMILIN1, FBN1, FSTL1, GLT8D2, LRRC32, MMP2, TGFB*, and *TIMP2*, cor.MM >0.8 and cor.GS >0.4) in the yellow module (Figure 3C).

### Functional Annotation of Modules

**Figure 4:**
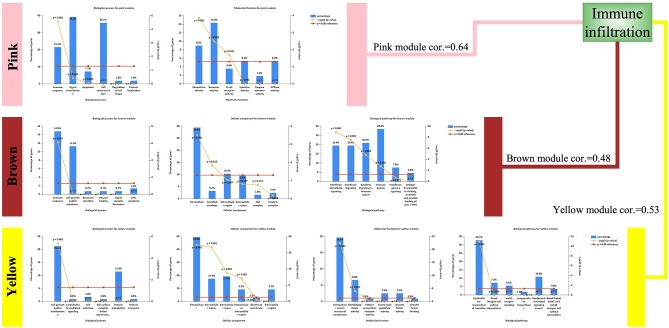
Enriched functions of the modules highly correlated with immune infiltration. Biological processes (BPs), cellular components (CCs), molecular functions (MFs), and biological pathways are listed. For detailed enrichment results, refer to the Supplementary Material.

Although the three modules had high correlations with immune infiltration, the functions were not the same (Figure 4). Both the pink module and the brown module were related to immune responses. The pink module was also related to chemokine activity, and the brown module was related to interferon signaling, both of which are immune-related functions. However, the yellow module was different, showing associations with the extracellular matrix and the epithelial–mesenchymal transition (EMT). Although this result was surprising, it was consistent with previous reports demonstrating that EMT-related genes are related to resistance to PD-1 blockade in bladder cancer (21).

### Cluster Analysis

**Figure 5:**
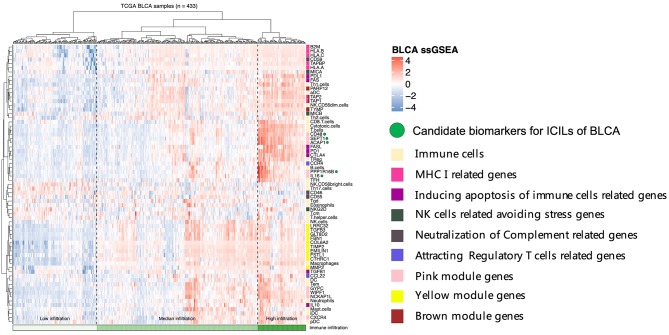
Five candidate biomarkers were found by unsupervised clustering, and the sources of genes identifying different immune function modules can be found in the Discussion section.

To assess the relationships among immune cells, immune-related genes, and key genes, unsupervised clustering was performed using ssGSEA (Figure 5). We noted that the genes from different modules were clustered in different zones that were close to each other. However, the key genes in the pink module were distributed in two cluster zones, suggesting that differences existed among these key genes, even though their biological functions were similar. *CD48, SEPT1*, and *ACAP1* in the pink module were strongly associated with the toxicity of T cells, whereas *PPP1R16B* and *IL16* also showed similar associations but were more closely related to B cells and Tfh cells. Among the key genes in the brown module, *TAP1* and *TAP2* encode components of MHC I. Together with *PARP12* and *TYMP*, these key genes were closely related to aDCs and NK CD56^dim^ cells. The key genes in the yellow module, which was related to the mechanisms of the EMT and extracellular matrix, were clustered even more closely together and had the closest relationship with NK cells. Based on the results of these cluster analyses, we considered *CD48, SEPT1, ACAP1, PPP1R16B*, and *IL16* in the pink module as important candidate genes because their expression levels best reflected the ICILs of the samples and cytotoxic function.

### TIMER Validation and Co-expression

**Figure 6:**
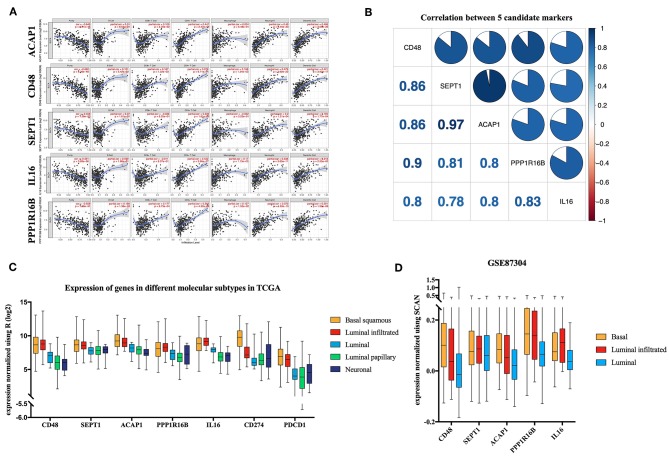
Verification of five candidate markers. **(A)** The correlation of five biomarkers with tumor purity and fraction of immune cells. **(B)** The five candidate biomarkers were highly correlated with each other at the transcript level. Correlation analysis using Spearman correlation; results with *P*-values of < 0.0001 were considered significant. **(C)** Candidate biomarkers were highly expressed in the basal squamous subtype and the luminal infiltrated subtype, and the ICILs of these two subtypes were the highest. Gene expression differed among subtypes (*P* < 0.01). Differences were analyzed by one-way ANOVA. **(D)** In GSE87304, the expression of the candidate genes in the previously described subtypes was still the highest.

TIMER utilizes data from the TCGA database to facilitate the visualization of associations among genes and immune infiltration. *CD48, SEPT1, ACAP1, PPP1R16B*, and *IL16* were all negatively correlated with tumor purity, supporting the results for highly correlating with ICILs (Figure 6A). Additionally, the co-expression of these five genes was analyzed using the R corplot package, and no correlations were <0.78; the correlation between *ACAP1* and *SEPT1* reached 0.97 (Figure 6B).

### Key Genes in Molecular Subtypes

There was a significant difference in the expression of the key genes in different molecular subtypes based on TCGA data, using one-way analysis of variance (ANOVA). Among the five molecular subtypes, five key genes were highly expressed in the basal and luminal infiltrated subtypes, both of which had low responses to IMCIs (Figure 6C). In Gene Expression Omnibus, GSE87304 with molecular subtype was screened out for key genes. The expression levels of the five key genes were higher in the basal and luminal infiltrated subtypes than in the luminal subtype (Figure 6D). The basal and luminal infiltrated subtypes had the highest ICILs and the worst IMCI response rate.

## Discussion

The use of IMCIs as therapeutic agents for BLCA has resulted in favorable outcomes in some patients; however, most patients do not benefit from treatment with these agents. Tumor immunity is an extremely complex biological process, and factors affecting the efficacy of IMCIs include PD-L1 expression levels and TMB. In our study, we found that ICILs affected the response rate of IMCIs, and higher ICILs led to lower response rates. In order to easily identify the ICILs of BLCA, the modules obtained by WGCNA were related to the ICILs calculated by ssGSEA, and key genes were obtained through further screening and unsupervised clustering. High expression of the key genes reduced the effectiveness of IMCI treatment.

Our results showed that increased expression of key genes indicated decreased tumor purity, which was not solely caused by ICILs because there are many other cells, such as cancer-associated fibroblasts (CAFs), also found in the tumor (22). CAFs can promote the EMT and are also associated with tumor progression and treatment resistance (23, 24). Although CAFs are likely to be associated with ICILs, the yellow module, which was enriched in EMT and extracellular matrix functions, was highly correlated with ICILs. Therefore, CAFs may be responsible for the high resistance of IMCIs in high ICILs.

In this study, we identified a number of representative genes related to immunity and found that these genes were involved in the immune system through different mechanisms. During the development of tumor immunity, tumors show many alterations, including suppression of identity characteristics and stress, induction of immune cell apoptosis, attraction of Tregs, and neutralization of complement. These processes may block the immune system from clearing tumor cells, and the genes involved in these processes are initially aggregated for subsequent cluster analysis. The process of hiding the tumor identity involves major histocompatibility complex (MHC) I-related genes (e.g., *HLA-A/B/C, TAP1/2, TAPBP*, and *B2M*). The genes that hide stress to prevent NK cells from attacking include *MICA, MICB*, and *NKG2D* (25). Additionally, genes involved in counterattacking immune cells and inducing apoptosis include *PD-1* (*PDCD1*), *PD-L1* (*CD274*), and *CTLA4* (26). FAS also binds to FASL to activate the non-inherent apoptotic pathway (27, 28), and tumor cells release transforming growth factor (TGF)-β and interleukin (IL)-10 to counterattack immune cells (29, 30). Tregs block lymphocytes from attacking tumor cells, which release C–C motif chemokine ligand 22 to bind to Tregs expressing C–C motif chemokine receptor 4, thereby attracting Tregs to tumors (31). CD46, CD55, and CD59 are membrane-bound complement regulatory proteins that prevent complement-mediated cytolysis (31, 32).

In our cluster analysis, we included genes that have been shown to be associated with ICILs. Among these genes, the well-known T-cell surface receptors PD-1 and CTLA4 (33) were clustered in the same group as the five important candidate genes: *CD48* (encoding CD48), *SEPT1* (encoding Septin 1), *ACAP1* (encoding ArfGAP with coiled-coil, Ankyrin repeat, and PH domains 1), *PPP1R16B* (encoding protein phosphatase 1 regulatory subunit 16B), and *IL16* (encoding IL-16). These genes were also closely related to CD8 T cells and cytotoxic cells. Moreover, in the final molecular subtype validation, their expression was highest in the basal and infiltrated subtypes of high ICILs. High expression of these molecules could indicate that the effects of IMCIs may decrease.

To elucidate the associations between these five genes and TILs, further investigations showed that except for *PPP1R16B*, all other genes had been previously reported to be associated with immune cells. CD48, a member of the signaling lymphocyte activation molecule family, participates in the adhesion and activation of immune cells, thereby contributing to T-cell activation and proliferation (34). SEPT1, a member of the septin family of GTPases, contributes to cancer progression and proliferation in oral squamous cell carcinoma (35). Septins are cytoskeletal proteins that provide compression and rigidity and support efficient motion of motile T cells (36). PPP1R16B (TIMAP), an endothelium-enriched TGF-β downstream protein that structurally belongs to the targeting subunit of myosin phosphatase, regulates macrophage M2 phenotypic phagocytosis (37). IL-16, a cytokine known for its chemotactic and inflammatory properties, induces proliferation in cutaneous T-cell lymphoma T cells and plasma cells in multiple myeloma and recruits CD4^+^ protumor macrophages in breast cancer (38, 39).

The brown module, which included the MHC components TAP1 and TAP2, and the yellow module, which was closely related to the mechanism of the EMT, were both associated with ICILs. Cytotoxic T cells are known to destroy cancer cells by recognizing the peptides presented via MHC-I on the tumor cell surface (40). In addition, the EMT not only is involved in tumor metastasis but also promotes immune escape and enhances immunosuppressive signals (41, 42). Therefore, ICILs in tumors are a manifestation of a very complex biological process. Our findings showed that high ICILs may have no advantage in the treatment of IMCIs.

In the calculation of the response rates of different ICILs for IMCIs, we showed that the response rates of different molecular subtypes in various ICILs were the same. This may have caused some bias in the results. However, in BLCA, few studies have evaluated IMCIs using sequencing or array profiling. Further studies are needed to verify our conclusions.

In summary, our results suggested that patients with high ICILs may benefit the least from treatment with IMCIs in BLCA. *CD48, SEPT1, ACAP1, PPP1R16B*, and *IL16* were candidate genes for determining ICILs in BLCA, and these genes may have application as biomarkers to guide treatment with IMCIs.

## Data Availability Statement

Publicly available datasets were analyzed in this study. These data can be found here: https://portal.gdc.cancer.gov.

## Author Contributions

BL proposed the study concept, design, and drafted the manuscript. BL and SP collected, analyzed, and interpreted the data. YZ, XC, and BW participated in revising the manuscript. All authors have read and approved the final manuscript.

### Conflict of Interest

The authors declare that the research was conducted in the absence of any commercial or financial relationships that could be construed as a potential conflict of interest.

## Supplementary Material

Supplementary Figure 1 **(A)** Clustering of samples and removal of outliers. **(B)** Analysis of network topology for various soft-thresholding powers in scale independence and mean connectivity.

Supplementary Table 1 Enrichment analysis of brown module.

Supplementary Table 2 Enrichment analysis of pink module.

Supplementary Table 3 Enrichment analysis of yellow module.
