# Supplementary figures and images for: Bladder Cancer Exhibiting High Immune Infiltration Shows the Lowest Response Rate to Immune Checkpoint Inhibitors
Source: Front Oncol. 2019 Oct 31;9:1101. doi: 10.3389/fonc.2019.01101 (PMC6834825; doi:10.3389/fonc.2019.01101)

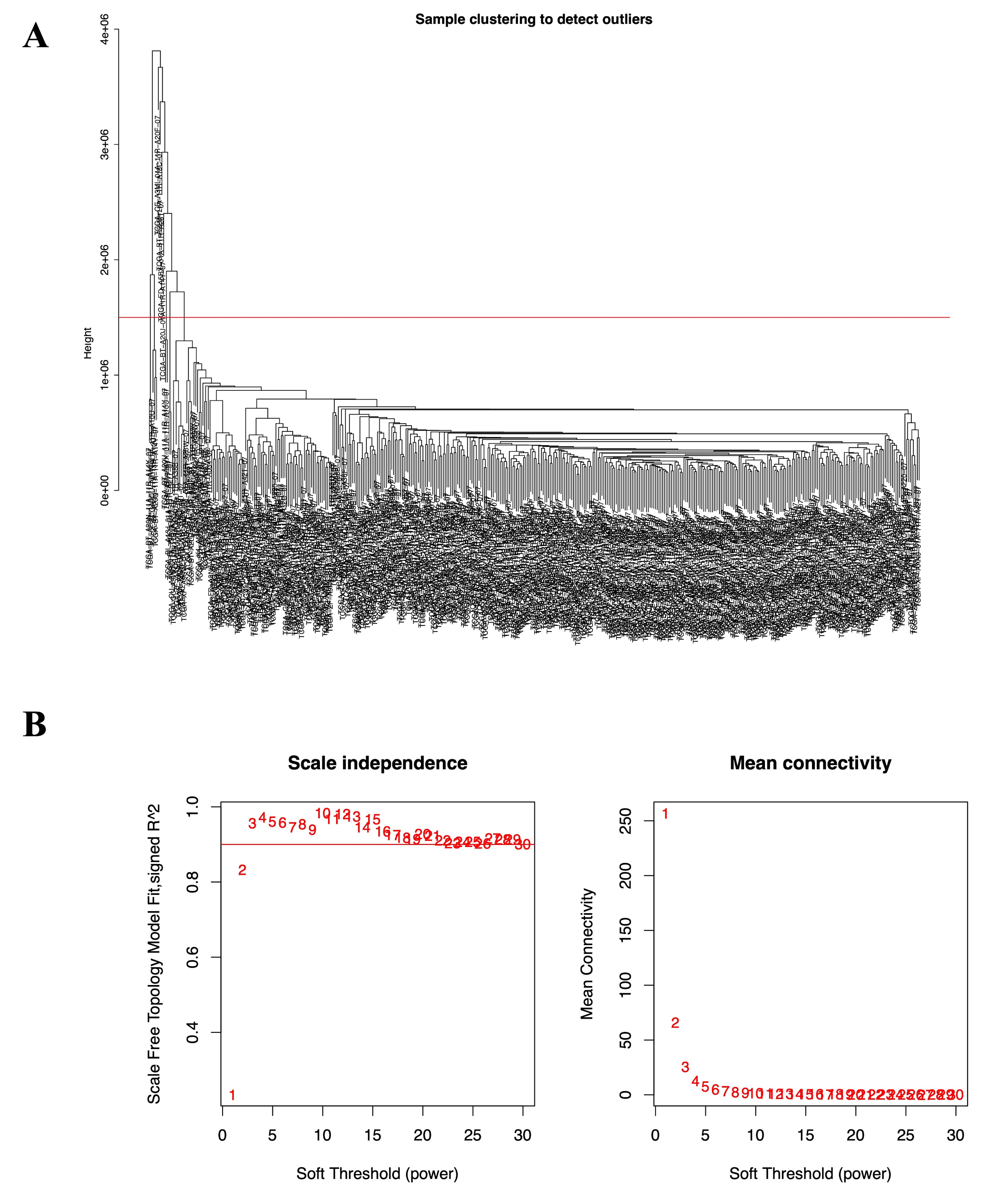

Supplement: Supplementary Figure 1 — (A) Clustering of samples and removal of outliers. (B) Analysis of network topology for various soft-thresholding powers in scale independence and mean connectivity. [file Image_1.JPEG]
